# Determining the prevalence of childhood hypertension and its concomitant metabolic abnormalities using data mining methods in the Northeastern region of Hungary

**DOI:** 10.3389/fcvm.2022.1081986

**Published:** 2023-01-10

**Authors:** Beáta Kovács, Ákos Németh, Bálint Daróczy, Zsolt Karányi, László Maroda, Ágnes Diószegi, Bíborka Nádró, Tamás Szabó, Mariann Harangi, Dénes Páll

**Affiliations:** ^1^Division of Metabolic Disorders, Department of Internal Medicine, Faculty of Medicine, University of Debrecen, Debrecen, Hungary; ^2^Institute for Computer Science and Control, Eötvös Loránd Research Network (ELKH SZTAKI), Budapest, Hungary; ^3^Université catholique de Louvain, INMA, Louvain-la-Neuve, Belgium; ^4^Department of Medical Clinical Pharmacology, Faculty of Medicine, University of Debrecen, Debrecen, Hungary; ^5^Department of Pediatrics, Faculty of Medicine, University of Debrecen, Debrecen, Hungary

**Keywords:** adolescents, blood pressure, children, data mining, hypertension, metabolic parameters, obesity, prevalence

## Abstract

**Objective:**

Identifying hypertension in children and providing treatment for it have a marked impact on the patients’ long-term cardiovascular outcomes. The global prevalence of childhood hypertension is increasing, yet its investigation has been rather sporadic in Eastern Europe. Therefore, our goal was to determine the prevalence of childhood hypertension and its concomitant metabolic abnormalities using data mining methods.

**Methods:**

We evaluated data from 3 to 18-year-old children who visited the University of Debrecen Clinical Center’s hospital throughout a 15-year study period (*n* = 92,198; boys/girls: 48/52%).

**Results:**

We identified a total of 3,687 children with hypertension (2,107 boys and 1,580 girls), with a 4% calculated prevalence of hypertension in the whole study population and a higher prevalence in boys (4.7%) as compared to girls (3.2%). Among boys we found an increasing prevalence in consecutive age groups in the study population, but among girls the highest prevalences are identified in the 12-15-year age group. Markedly higher BMI values were found in hypertensive children as compared to non-hypertensives in all age groups. Moreover, significantly higher total cholesterol (4.27 ± 0.95 vs. 4.17 ± 0.88 mmol/L), LDL-C (2.62 ± 0.79 vs. 2.44 ± 0.74 mmol/L) and triglyceride (1.2 (0.85-1.69) vs. 0.94 (0.7-1.33) mmol/L), and lower HDL-C (1.2 ± 0.3 vs. 1.42 ± 0.39 mmol/L) levels were found in hypertensive children. Furthermore, significantly higher serum uric acid levels were found in children with hypertension (299.2 ± 86.1 vs. 259.9 ± 73.3 μmol/L), while glucose levels did not differ significantly.

**Conclusion:**

Our data suggest that the calculated prevalence of childhood hypertension in our region is comparable to data from other European countries and is associated with early metabolic disturbances. Data mining is an effective method for identifying childhood hypertension and its metabolic consequences.

## 1. Introduction

Childhood hypertension presents a considerable public health challenge worldwide, as it is a potent risk factor for adult hypertension with predictive values varying between 19 and 65% (1). Indeed, it is thought that the origin of the onset of hypertension in adults stems from childhood (tracking phenomenon) (2). The number of children diagnosed with hypertension has significantly grown in the past few decades. The rise in numbers is associated mainly with the obesity epidemic and in part with high salt intake, which may not be related to the economic status of a given country (3). Hypertension is common in adolescents undergoing puberty and in children who are overweight or obese. Some further factors increase the risk of primary hypertension, such as low birth weight, male sex, a sedentary lifestyle, a family history of hypertension and African-American ethnicity (4). According to recent data, primary hypertension is now the leading form of hypertension in childhood, especially in adolescents (5). Adolescents usually have primary hypertension, making up 85 to 95% of cases. Childhood hypertension, particularly in preadolescents, can be secondary to an underlying disorder. The parenchymal disease of the kidneys is the most common secondary cause of hypertension in early childhood. Endocrine diseases contribute to elevated blood pressure. Pheochromocytoma, hyperthyroidism, hyperaldosteronism and the impact of pharmaceuticals (e.g., oral contraceptives, sympathomimetics and some dietary supplements) can be cited as well (6).

The universal screening of hypertension in childhood needs to be improved, as its low prevalence leads to the misdiagnosis of childhood hypertension (7). Furthermore, identifying arterial hypertension is challenging in children and adolescents since standards and definitions are complex during body growth. Therefore, designing cardiovascular outcome studies becomes a challenge, too.

Due to the different positions of current guidelines, the global definition of hypertension in children and adolescents is precarious. Therefore, evaluating the prevalence of hypertension in this age group is complicated on a global scale (8). In Europe, the Scientific Council and the Working Group on Hypertension in Children and Adolescents of the European Society of Hypertension (ESH) updated its 2009 guidelines in 2016 (9).

A former cross-sectional, population-based study conducted in a Hungarian city (Debrecen, population 230,000) found that more than two decades ago the prevalence of hypertension was 2.53% in adolescents (15-18 years of age) (10). Other studies show that the most probable prevalence of childhood and adolescent hypertension is estimated to be 3.5% (11, 12). A recently published random-effects meta-analysis of 47 articles estimates the pooled prevalence of childhood hypertension to be 4.00% (95% CI, 3.29-4.78%) (13). Screening for childhood hypertension in Eastern Europe is relatively poor despite the fact that the findings of a large number of studies are available. We aim to identify childhood hypertension in our region by evaluating a period of 15 years using data mining methods to analyze a rather large population. Although this method is not widely used in epidemic studies, it is an excellent way to define prevalence in large patient cohorts. To date, this is the first paper providing data on childhood hypertension in Europe to embrace the benefits of data mining methods. A large pediatric patient population is selected to calculate the estimated prevalence of hypertension and some significant concomitant diseases and metabolic parameters.

## 2. Patients and methods

### 2.1. Screening patients for hypertension

As we have delineated previously in other studies of ours (14–16), various methods of data mining used on mass hospital data are ideal for screening for medical diagnoses in cases of hypertension and other conditions. The clinical diagnosis of hypertension was based on the competence of our highly educated pediatricians. Most of them were specialists in endocrinology and nephrology at our University Center. They precisely followed the available international guidelines. According to the current recommendations, they define hypertension by three consecutive elevated blood pressure readings - the measured values compared to age-specific reference values. Our specialists used devices validated for clinical accuracy. The diagnosis of hypertension was based on textual history data (phrases hypertension and its synonyms) and diagnosis codes for hypertension (International Statistical Classification of Diseases and Related Health Problems 9th and 10th Revision, WHO) recorded in the source data.

*Via* the University of Debrecen Clinical Center we have gained access to anonymous medical records compiled in the Northeastern region of Hungary for the purpose of software development. The source data included the totality of medical records from the clinical centre from a period of 1 January 2007 to 31 December 2021. Our team and our cooperating partner, Black Horse Group Ltd., launched a data mining project wherein we were permitted to make use of their medical system framework entitled “AescuLab”^1^. Data extracted from the clinical records underwent a multistep procedure of anonymization for purposes of protecting patients’ private particulars, while the tables of specific case and patient data were detached from real persons. We made use of open-source tools such as https://numpy.org/; http://pandas.pydata.org/ well as certain scripts and solutions developed on our own for cleaning data and completing missing or corrupt sections of data, compiling a totalized and integrated data source from isolated data with laboratory cases, anamneses, national diagnosis codes as well as statistical data on the patients. Our serializing and buffering methods ensured that the data as processed to ward off problems that might be taken for granted in the case of such an enormous data source. As a pre-processing step, textual information was processed through parsing and stemming^2^, bag-of-words (BOW) modeling. We ranked phrases by the “Term Frequency/Inverse Document Frequency” (TF-IDF) method (17) and carried out word2vec (W2V) modeling in Keras^3^ in order to spot key role phrases (18). When creating BOW models, documents are outlined as histograms of reoccurring terms/words but the models do not take into account any sequential structure, resulting in the representation being vigorous and invariant where documents would consist of deviating elements of sequences. Further, using W2V models ensures that terms and phrases are described as elements in a vector space defined by a neural network, a straightforward language model using contextual terms to identify sequential elements utilizing the structure of the sequence. These two models are equally efficient in determining the significance of words/expressions by ranking them on the basis of their IDF score (17) or perplexity (18). In addition, we compiled a term list using professional vocabularies and utilized string-matching algorithms to exert control over misspellings and to recover the corrupted terminology from fragmented data.

Some of the data that we extracted and processed contains normal anamneses which have not yet undergone any processing and thus necessitate the use of pre-processing methods such as text extraction and content identification focusing on regular terminology. The resulting data yields a finite set full of phrases with the number of occurrences per document recorded, with another value added where previous medical checkups were conducted. Initially our term list ran several million items, but using the aforementioned methods yielded a much more accessible list of 250,000 phrases. Linking cases, patient records and diagnoses helped to display the patients’ medical histories as temporal sequences of events closely linked to the patients, which format facilitated the pinpointing of patients with hypertension.

The work complies with the guidelines of the Declaration of Helsinki. The protocol was approved by local and regional ethical committees.

### 2.2. Determining cardiovascular risk factors amongst laboratory settings

As the data sets compiled were based in various different data structures, the initial step was to establish joint representation to facilitate statistical analysis. This type of structure is necessary to ensure the detection of specific ‘attributes’. For instance, high blood pressure might crop up in the text-based data in the guise of various different words, as a parameter or derivative of real measurements. Different data extraction tools were developed and applied to the source data. Another challenge we faced was data cleansing, as we had to complete missing or corrupt fragments of data, where each type of corrupted data had to be treated in a different manner. For instance, the deployment of gap-stopping binary variables with mean values is a method that is ambiguous and thus it had to be eschewed, so all such individual cases were interpreted as normal phrases. In the light of the sheer magnitude of data, further serializing and streaming techniques were developed to optimize the final query engine, which is able to manage partial data and might identify attributes utilizing deduction. These included parsing, stemming, the filtering of stop-words and building dictionaries from unigrams and bigrams after the phrases/words were cleaned manually. Utilizing the TF-IDF (term frequency-inverse document frequency) and word embeddings techniques we found data with phrases and Word embeddings in Hungarian were trained on traditional corpora, which necessitated compiling our language model using the available text as data. We established a Gated Recurrent Unit model (19), a recurrent neural network where cleaned unigrams and bigrams were compiled as dictionaries. Our output data structure appeared thus: an ‘attribute’ was linked to a patient upon the existence of one of certain specific events. A regular expression was identified or had a high probability where on the basis of the language model it was a phrase or emerged as data from laboratory measurements.

### 2.3. Laboratory analysis

We measured routine laboratory parameters, including glucose, urea, creatine and uric acid from fresh sera using a Cobas c501 analyzer (manufactured by Roche Ltd., Mannheim, Germany) in the Laboratory Medicine Unit, Clinical Centre, University of Debrecen. The measurement of total cholesterol levels is performed through enzymatic and colorimetric tests (cholesterol oxidase-p-aminophenazone—GPOD-PAP; Modular P-800 analyzer; Roche/Hitachi). The measurement of HDL cholesterol and LDL cholesterol levels was carried out using a homogenous enzymatic and colorimetric assay (Roche HDL-C as well as third generation for HDL cholesterol and Roche LDL-C plus second generation for LDL cholesterol). We performed tests according to the recommendations of the manufacturer.

### 2.4. Statistical analysis

Recorded anonymous patient data from the University of Debrecen Clinical Center’s clinical IT system was used. We gained access to the data source in HL7 format, partially cleaned and pre-processed by the university’s partner company, Aesculab Medical Solutions, Black Horse Group Ltd., who cleaned the data to be used for their data mining and machine learning objectives. Leveraging the database at the outset ensured the avoidance of system errors which might have resulted from the manner in which the original clinical data was recorded, spanning 15 years (from 2007 to 2021), containing the entirety of the clinical centre’s patient record database with all the textual, diagnostic and laboratory particulars. We extracted the data through queries from the PostgreSQL 13.x database, which yielded enormous text files, which were then used as a kickoff for subsequent statistical analysis. The population involved in the study included the number of patients treated at the University of Debrecen throughout this period, totaling 92,198 persons, whose data were derived from all departments and all inpatient and outpatient information sources available from the above specified time period.

Statistical analysis was carried out through the deployment of Python-supported data mining packages. Data cleaning and processing were performed by utilizing Python 3.8, IPython 7.29, Cython 0.29, Pandas 0.23 and Numpy 1.22 under Conda 4.10 environment with Dask. Machine learning applied to refine data selection and perform deep textual analysis leveraged SciKit-Learn 1.0 and Pytorch 1.09. Unpaired t-tests conducted for statistical significance analysis maintained a significance level of 95%. We created statistical figures with the MatPlotLib 3.5 software package.

## 3. Results

**FIGURE 1:**
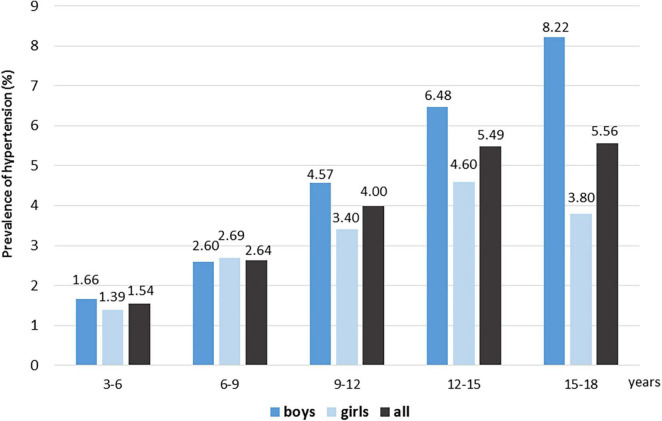
Prevalence of hypertension in different age and gender groups.

Based upon the data source containing laboratory cases, textual history data, diagnosis codes and patient statistic data, we evaluated each child from ages 3 to 18 years that visited the University of Debrecen Clinical Center’s hospital during the 15-year study period (total number of 3 to 18-year-old children *n* = 92,198; boys/girls: 44,380/49,084; 48/52%). We identified a total of 3,687 children with hypertension (2,107 boys and 1,580 girls), which means that the calculated prevalence of hypertension in the whole study population is 4%, a higher prevalence in boys (4.7%) as compared to girls (3.2%). We divided the study population into five age groups (3-6 years; 6-9 years, 9-12 years, 12-15 years and 15-18 years). The 3-6 year-old age group was defined as the period above the age of 3 years and below the age of 6 years. We found an increasing prevalence in the consecutive age groups in the whole study population (1.54; 2.64; 4.0; 5.49 and 5.56%, respectively), and in the boys (1.66; 2.6; 4.57; 6.48 and 8.22%), while in girls the highest prevalence was identified in the 12-15-year-old age group (1.39; 2.69; 3.4; 4.6 and 3.8% was found, respectively) (Figure 1).

**FIGURE 2:**
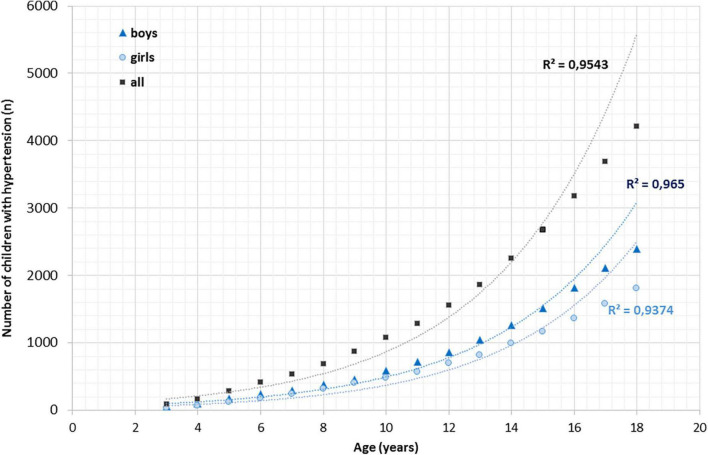
Exponential correlation between age and cumulative number of children with hypertension.

We found a strong linear correlation between age and the number of children diagnosed with hypertension in the whole study population (R2 = 0.93), in boys (R2 = 0.91) and in girls (R2 = 0.93). Likewise, an exponential correlation was detected between the cumulative number of children in various age groups diagnosed with hypertension in the whole study population (R2 = 0.97), in boys (R2 = 0.98) and girls (R2 = 0.96) (Figure 2).

**FIGURE 3:**
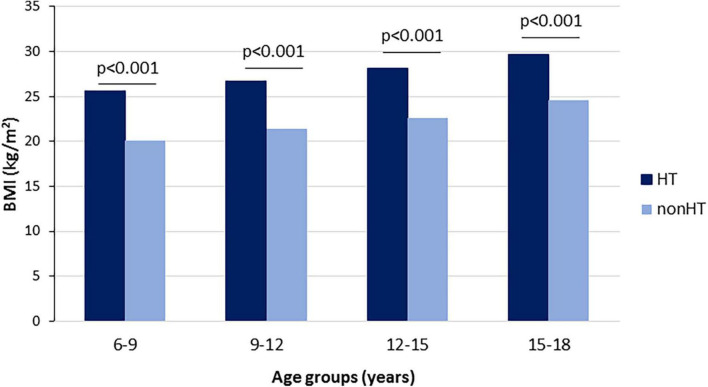
Average body mass index (BMI) values in children with and without hypertension in different age groups.

We also evaluated the prevalence of the most significant concomitant diseases. We found a markedly and significantly higher prevalence of obesity (49.7 vs. 6.16%), diabetes mellitus (7.17 vs. 1.27%), renal diseases (2.81 vs. 0.51%) and thyroid diseases (8.74 vs. 6.22%) in hypertensive children as compared to normotensives. Obesity was the most prevalent concomitant disease in all patient groups, with the highest prevalence documented in the 6-9-year-old and the 9-12–year-old age groups (65%). Moreover, we found higher BMI values in hypertensive children as compared to the normotensive group in all studied patient groups (25.6; 26.7; 28.1 and 29.7 vs. 20.1; 21.4; 22.6 and 24.6 kg/m^2^ in the 6-9–year-old, 9-12–year-old, 12-15–year-old and 15-18–year-old groups, respectively) (Figure 3).

**FIGURE 4:**
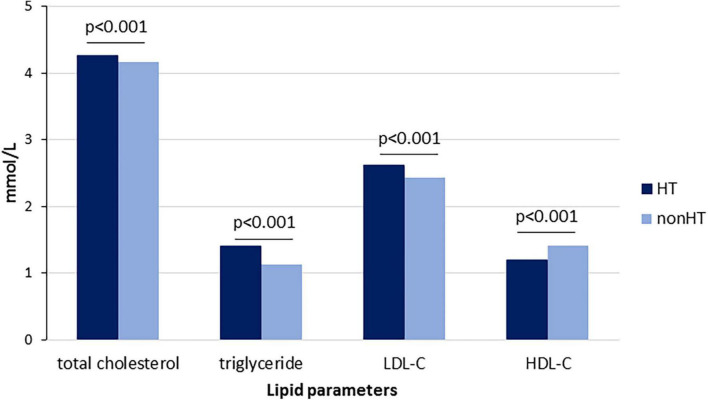
Serum lipid parameters in children with and without hypertension in the whole patient population.

Table 1 lists the laboratory parameters. We found significantly higher total cholesterol (4.27 ± 0.95 vs. 4.17 ± 0.88 mmol/L), LDL-C (2.62 ± 0.79 vs. 2.44 ± 0.74 mmol/L) and triglyceride (1.2(0.85-1.69) vs. 0.94(0.7-1.33) mmol/L), and lower HDL-C (1.2 ± 0.3 vs. 1.42 ± 0.39 mmol/L) levels in children with hypertension compared to the normotensive children in the whole study population (Figure 4) and in all age groups (Table 1). Except for the first age group, total cholesterol level was higher in hypertensive children compared to the normotensive group. Furthermore, significantly higher serum uric acid levels were found in children with hypertension (299.2 ± 86.1 vs. 259.9 ± 73.3 μmol/L). There were no reportable differences in glucose, urea and creatine levels between the hypertensive and normotensive groups throughout the various age groups.

**TABLE 1:** Laboratory parameters of the study population.

	3-6 ys		6-9 ys		9-12 ys		12-15 ys		15-18 ys	
	**HT**	**Non-HT**	**HT**	**Non-HT**	**HT**	**Non-HT**	**HT**	**Non-HT**	**HT**	**Non_HT**
N	195 (68.7%)	14756 (80.5%)	300 (74.3%)	12699 (85.9%)	409 (68.9%)	11747 (82.4%)	704 (72.6%)	13346 (79.9%)	978 (68.3%)	18856 (77.5%)
Glucose (mmol/L)	**4.74 ± 0.81**	**5.02 ± 1.72**	5.05 ± 3.4	5.05 ± 1.44	**4.90 ± 1.17**	**5.13 ± 1.7**	5.07 ± 1.52	5.16 ± 1.43	5.09 ± 1.2	5.06 ± 1.19
TC (mmol/L)	**4.08 ± 1.05**	**4.14 ± 0.99**	**4.25 ± 0.90**	**4.12 ± 0.82**	**4.31 ± 0.94**	**4.14 ± 0.82**	**4.23 ± 0.93**	**4.12 ± 0.81**	**4.30 ± 0.97**	**4.24 ± 0.92**
LDL-C (mmol/L)	**2.52 ± 0.77**	**2.50 ± 0.88**	**2.60 ± 0.74**	**2.44 ± 0.74**	**2.68 ± 0.79**	**2.43 ± 0.71**	**2.58 ± 0.79**	**2.39 ± 0.65**	**2.64 ± 0.82**	**2.46 ± 0.76**
HDL-C (mmol/l)	**1.2 ± 0.31**	**1.38 ± 0.36**	**1.21 ± 0.32**	**1.39 ± 0.39**	**1.18- ± 0.28**	**1.39 ± 0.37**	**1.19 ± 0.3**	**1.42 ± 0.38**	**1.22 ± 0.31**	**1.46 ± 0.41**
TG (mmol/L)	**1.2 (0.78-1.56)**	**1.0 (0.7-1.4)**	**1.14 (0.83-1.7)**	**0.93 (0.69-1.3)**	**1.3 (0.9-1.76)**	**0.98 (0.7-1.41)**	**1.19 (0.85-1.6)**	**0.94 (0.7-1.3)**	**1.13 (0.85-1.64)**	**0.9 (0.68-1.3)**
Uric acid (μ mol/L)	**241 ± 67.4**	**228 ± 59.8**	**259 ± 73.1**	**247 ± 66.9**	**283 ± 82.3**	**262 ± 72.6**	**309 ± 90.3**	**275 ± 73.4**	**316 ± 82.4**	**273 ± 76.7**
Urea (mmol/L)	4.1 (3.5-5.0)	4.03 (3.4-4.7)	4.13 (3.5-4.9)	4.05 (3.5-4.8)	4.2 (3.5-4.8)	4.1 (3.5-4.8)	4.18 (3.6-4.9)	4 (3.4-4.7)	4.2 (3.6-5.0)	4.0 (3.3-4.8)
Creatine (μ mol/L)	31.5 (26.2-38)	32 (27-40)	39 (33-46.3)	43 (35-53)	46 (39-55.5)	50 (42-61.5)	57 (47.1-66.4)	56 (49-66)	63 (54-74)	62 (53-72)

Values are presented as mean ± standard deviation or median (lower quartile - upper quartile). Number of patients (n) indicates the number of cases with available laboratory data (percent compared to the complete study group). Significant differences (*p* < 0.05) between the study groups are marked with bold letters. HDL-C, high-density lipoprotein-cholesterol; HT, hypertensive; LDL-c, low-density lipoprotein cholesterol; non-HT, non-hypertensive; TC, total cholesterol; TG, triglyceride.

The prevalence of several secondary causes resulting in hypertension such as hyperthyroidism, hyperaldosteronism, Cushing syndrome, phaeochromocytoma, hyperparathyroidism and chronic renal disease was a shade higher in the most hypertensive age groups, but because of the scarcity of cases, we could not perform statistical analysis. Indeed, the documentation of rare causes of secondary hypertension appears to be insufficient and inaccurate.

## 4. Discussion

Data mining techniques gained ground in clinical diagnostics, and we can use them for several purposes, including research in the biomedical and healthcare fields (20). Since data on childhood hypertension in the Eastern European regions are scarce, and we have successfully used data mining methods for screening some rare hereditary dyslipidemias previously (14, 16), we aimed to investigate a 15-year period and a large pediatric patient population to calculate the estimated prevalence of childhood hypertension and its concomitant diseases using data mining methods. Our data suggest that the calculated prevalence of childhood hypertension in Hungary, at least in our region, is 4%. It is comparable to the data of other European countries and is in line with the result of a recent meta-analysis (13). In agreement with the literature data, the prevalence of hypertension increases with age from 1.54% up to 5.56%, showing gender differences and a markedly higher ratio of hypertension in boys. In adolescent boys (from 15 to 18 years), the prevalence of hypertension was more than twice the average (8.22%), highlighting the importance of screening in this subpopulation.

As far as hypertension etiology is concerned, the traditional separation is primary and secondary classification. Primary hypertension in children is mainly hereditary. The inheritance pattern is multifactorial and modified by environmental factors and diet, such as highly processed food and sodium intake (21). Although we could not extract data on these parameters, a recent study reported that sodium intake exceeds while potassium does not reach dietary recommendations in Hungary (22). A multicenter paper proved that almost half of the daily energy intake of children from eight European countries including Hungary came from ultra-processed foods (23). A significant connection between obesity and hypertension is well-documented among children. Our data also highlight the importance of obesity in this patient population: we found significantly higher BMI values and markedly higher obesity prevalence in hypertensive children. As in adults, obesity due to poor diet and inactivity is the most important predisposing factor for metabolic abnormalities in childhood, including dyslipidemia, hyperinsulinemia and hyperuricemia (24). Insulin resistance and hyperinsulinemia might contribute to enhanced renal sodium reabsorption or increased sympathetic nervous system activity (25). We found that the prevalence of diabetes was significantly higher among children with hypertension in all age groups. The impact of diet components on gene expression or fructose intake on uric acid levels also contributes to harmful metabolic changes (26). Although we have no data on diet components and fructose intake of the study population, serum uric acid levels were significantly higher in the hypertensive groups compared to the normotensive children. A former study found a positive association between uric acid and blood pressure, insulin and triglycerides in overweight and obese youths (27). We also found significantly higher serum triglyceride levels in all hypertensive age groups accompanied by lower HDL-C and higher LDL-C levels, indicating the complex disturbance of lipid metabolism. While dyslipidemia is common in adulthood, especially in overweight and obese population, its early appearance associated with childhood hypertension is astonishing and alarming, highlighting the importance of immediate laboratory screening at the moment of recognition. Although the pharmacological management of childhood dyslipidemia should be reserved for special situations, lifestyle intervention can be indicated at any age.

In secondary hypertension, there is an identifiable cause in the background. Secondary hypertension is relatively common in infants and young children (28). The causes of hypertension vary with age. Renal artery thrombosis or stenosis, congenital renal malformations, coarctation of the aorta or various endocrinological disorders can be the underlying cause of hypertension, although renal abnormality is the leading problem (21). Our data also demonstrated a higher prevalence of renal diseases in hypertensive children. Although we could not identify children with renal artery occlusion/stenosis or aortic coarctation, we found that the prevalence of several endocrine disorders was significantly higher in the hypertensive groups indicating their possible pathogenic role in hypertension. In adolescent females taking oral contraceptives is associated with hypertension. Unfortunately, data on the medication of adolescents enrolled in the study is not accessible.

Our study is not without some limitations. Hospitalgoers, both children and adults, represent a population that differs from the average population. Therefore, our calculations might overestimate the prevalence of hypertension, for example, the frequency of some secondary forms. The University of Debrecen Clinical Center is a regional centre for pediatric endocrinology and nephrology care that provides specialized, multidisciplinary care to children and adolescents with endocrine and renal disorders. We studied a relatively large cohort of children, which may not directly represent the total pediatric population in our region but may provide data. Unfortunately, we were unable to assess data on family history, diet and lifestyle habits. Additionally, a larger population is needed to define the contribution of secondary causes leading to childhood hypertension since their recording in medical documentation is precarious and inaccurate. Furthermore, we also collected data on patients’ antihypertensive medication. However, we could find only a few children treated with antihypertensive agents. In general, pediatricians administered beta-blockers and ACE-inhibitors in severe cases but primarily suggested lifestyle modification to the patients and their parents. We could not provide statistical data due to the low number of cases. Still, we believe that our data mining method verified their impact on the diagnostic process of childhood hypertension.

The rising prevalence of pediatric hypertension carries problematic global health dimensions. Childhood hypertension is no longer a condition characterized by elevated blood pressure values but rather a chronic disease associated with metabolic complications at presentation. Therefore, timely screening and interventions for these early metabolic complications are essential to prevent morbidity and mortality in the future. Hence, there is a pressing need for comprehensive pan-European action to increase knowledge on the prevention, diagnosis and treatment of high blood pressure in children and adolescents. To provide answers to questions and challenges, a multidisciplinary network was established recently, maintained and funded by the European Cooperation in Science and Technology (COST) Association, which will promote coordinated and collaborative activities on personalized preventive measures for children and adolescents across Europe (29). Till then, national datasets may contribute to our knowledge of the prevalence and characteristics of childhood hypertension.

## Data availability statement

The original contributions presented in this study are included in the article/supplementary material, further inquiries can be directed to the corresponding author.

## Author contributions

DP and MH: the study design. ÁN, BD, and ZK: development of methodology. ÁN, BD, and BN: collection of data. DP, MH, ÁD, ÁN, and LM: analysis and/or interpretation of data. DP, BK, and MH: writing (not revising) all or sections of the manuscript. TS: manuscript review. All authors contributed to the article and approved the submitted version.

## Footnotes

1
www.aesculab.net

2
http://hunspell.github.io/

3
https://keras.io/
